# Long-term impacts of rising sea temperature and sea level on shallow water coral communities over a ~40 year period

**DOI:** 10.1038/s41598-019-45188-x

**Published:** 2019-06-19

**Authors:** B. E. Brown, R. P. Dunne, P. J. Somerfield, A. J. Edwards, W. J. F. Simons, N. Phongsuwan, L. Putchim, L. Anderson, M. C. Naeije

**Affiliations:** 1 School of Natural and Environmental Sciences, Newcastle University, Newcastle upon Tyne, NE1 7RU UK; 2 Environmental Research Unit, University of the Highlands and Islands, Castle Street, Thurso, Caithness, KW14 7JD Scotland UK; 3 West Briscoe, Baldersdale, Barnard Castle, Co. Durham, DL12 9UP UK; 4 Plymouth Marine Laboratory, Prospect Place, West Hoe, Plymouth PL1 3DH UK; 5 Department of Space Engineering, Delft University of Technology, NL - 2629 HS Delft, Netherlands; 6 Department of Marine and Coastal Resources, 120 Moo 3, Changwathana Road, Bangkok, 10210 Thailand; 7 Phuket Marine Biological Center, PO Box 60, Phuket, 8300 Thailand; 8 Faculty of Biological Sciences, University of Leeds, Leeds, LS2 9JT UK

**Keywords:** Ecology, Climate sciences

## Abstract

Effects of combined rising sea temperature and increasing sea level on coral reefs, both factors associated with global warming, have rarely been addressed. In this ~40 y study of shallow reefs in the eastern Indian Ocean, we show that a rising relative sea level, currently estimated at ~11 mm y^−1^, has not only promoted coral cover but also has potential to limit damaging effects of thermally-induced bleaching. In 2010 the region experienced the most severe bleaching on record with corals subject to sea temperatures of >31 °C for 7 weeks. While the reef flats studied have a common aspect and are dominated by a similar suite of coral species, there was considerable spatial variation in their bleaching response which corresponded with reef-flat depth. Greatest loss of coral cover and community structure disruption occurred on the shallowest reef flats. Damage was less severe on the deepest reef flat where corals were subject to less aerial exposure, rapid flushing and longer submergence in turbid waters. Recovery of the most damaged sites took only ~8 y. While future trajectories of these resilient reefs will depend on sea-level anomalies, and frequency of extreme bleaching the positive role of rising sea level should not be under-estimated.

## Introduction

Recent literature has highlighted the climatic challenges facing corals in the 21^st^ century from thermally-induced bleaching^1,2^ and increasing sea levels^3^. Rising sea temperatures increase the threat of more frequent and intense bleaching events^4,5^, leading to coral mortality, with the forecast of annual bleaching becoming the future norm for many parts of the tropical seas^1,5^. In addition, it has been predicted that many coral reefs affected by severe bleaching where there is lack of recovery will not be able to keep up with sea-level rise under a Representative Concentration Pathway (RCP) 4.5 scenario^3^. Such a pathway is considered as an intermediate mitigation scenario where total radiative forcing is stabilised shortly after 2100 without overshooting the long-term radiative forcing target level^6^. For the Indian Ocean under RCP 4.5 sea level is projected to rise between 14–72 cm (mean 47 cm or 5.6 mm y^−1^) by 2100^3^. Following a bleaching event in 2016, reefs in the Seychelles and Maldives are reported to be in a negative accretion state and unlikely to be able to keep up with this projected sea-level rise unless rapid recovery takes place^3^. Indeed, it has been argued that bleaching events are now occurring too frequently to allow full recovery of reef assemblages^1^ which, in turn, would compromise reef accretion ability. While much of the interest in bleaching incidence and accretion potential of reefs in the Indian Ocean centres on the western and central sectors, little attention has been paid to reefs in the eastern basin. Here, due to a combination of physical and biological factors, corals in both shallow and deeper water settings appear to have been more resistant to repeated bleaching events over the last 27 years than their counterparts in the rest of the Indian Ocean^7^.

Such resistance has been shown by both offshore and inshore reefs in the Andaman Sea (north-eastern Indian Ocean)^8–10^. For offshore reefs, corals which are subjected to the effects of large amplitude waves show reduced bleaching and mortality^8,9^. In this environment the corals benefit from pulses of cool, nutrient rich water and also from high frequency temperature variability which has been shown to reduce the incidence of coral bleaching^11^. For shallow, inshore areas that include extensive macro-tidal reefs, thermally-induced bleaching is ameliorated by high seawater turbidity (and thus reduced ambient light intensity), extreme temperature variability, and remarkable physiological tolerances of the corals^7^.

At sites on the south eastern tip of Phuket in Thailand, macro-tidal fringing reefs have been intensively monitored for almost 40 y from 1979 to present. During this time there have been repeated thermally-induced bleaching events of varying severity in 1991, 1995, 1998, 2003, 2010 and 2016, with the 2010 bleaching being the most severe on record^10^. These reefs appear to have been seriously affected only by the 2010 bleaching event though they have also been subject to a variety of man-made and natural disturbances over time including major dredging activities for a deep water port in 1986–87^12^, prolonged negative sea-level anomalies induced by a marked Indian Ocean Dipole (IOD) in 1997^13^, and rising sea level as a consequence of climate change together with land subsidence following the 2004 Sumatran earthquake^14^.

Recovery from the earlier dredging and negative sea-level anomalies was rapid in both cases. More recently, although the 2010 bleaching episode resulted in a dramatic reduction in live coral cover, in the subsequent eight years luxuriant growth has led to a steady recovery in cover. Our earlier work^14^ demonstrated the beneficial effect of periods of elevated sea level on coral growth and has led us to hypothesise that long-term rising sea level might also be an important factor contributing both to coral recovery and also in providing protection to corals on the deeper reef flats during periods of bleaching. This paper tests this hypothesis by comparing data from our high-resolution long-term reef monitoring with records of sea surface temperature (SST), sea level, and vertical land movement.

## Methods

The study area is based on the SE tip of Ko Phuket, Thailand (8°00′N 98°20′E) (see Supplementary Information Fig. S1 for location). Reefs at this location are characterised by wide, sheltered reef flats that extend up to 200 m from the shore where they terminate in a shallow fore-reef extending to a depth of 5 m. All the reef flats have the same aspect and are subject to a similar hydraulic energy regime. The tides are semi-diurnal with a range of 0.6 m (neap tides) to 3.1 m (spring tides) with relatively little variation between successive high and low waters^14^. Data for coral cover and bleaching status have been collected semi-continuously from 1979 to 2018 (the ‘Study Period’).

### Environmental parameters

#### Sea Surface Temperature (SST) and Degree Heating Weeks (DHW)

**Figure 1:**
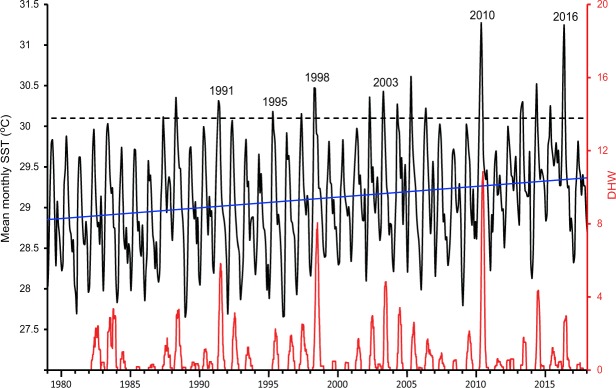
Mean monthly sea surface temperature (SST) and Degree Heating Weeks (DHW) from 1979–2017 for Phuket region. SSTs based on the UK Met Office HadSST3 dataset and DHW on values above a bleaching threshold of 30.1 °C (dashed line) based on the daily 0.25 degree gridded NOAA Optimum Interpolation Sea Surface Temperature (OISST) v2 high resolution dataset. Years when significant bleaching was recorded (see Table 1) are labelled. Solid blue line shows linear trend in SST over time (0.159 ± 0.03 °C (mean ± SE) per decade, GLS AR3 model (p < 0.0001).

The UK Met Office Hadley Centre’s 5°x5° gridded sea surface temperature, HadSST3 dataset of monthly SST anomalies^15,16^ was found to adequately reflect the inter-annual pattern of local calibrated *in-situ* thermistor sea temperature data (available for limited periods) and was thus used to derive the long-term pattern of SST (Fig. 1). The HadSST3 dataset employs 100 realisations with varying inputs to the bias adjustment algorithm to give a measure of the uncertainty. To derive the mean monthly SST climatology we used the mean value from the 100 realisations of the 1961–1990 monthly climatology^17^ for the Phuket area (grid-square centred on 7.5°N 97.5°E) and added the median SST anomaly for each month in the 1979–2018 study period. Data were extracted from NetCDF files and processed using Unesco-Bilko remote sensing image processing software (http://www.bilko.org/noaa_sst.php). Trend analysis was run in R 3.4.2^18^ using the package nlme^19^.

Degree Heating Weeks (DHW) for 1982–2017 were calculated using the daily 0.25 degree gridded NOAA Optimum Interpolation Sea Surface Temperature (OISST) v2 high resolution dataset (commencing September 1981) which combines observations from AVHRR satellites, ships and buoys. Data for the Phuket area (grid-square centred on 7.625°N 98.375°E) were extracted using Unesco-Bilko. Daily SST values exceeding a putative 30.1 °C bleaching threshold for Phuket^4^, were accumulated over a 12-week moving window to give a Degree Heating Week measure^20^ of relative thermal stress among years.

#### Sea-level

The fringing reefs at Phuket have been subject to the effects of rising sea level due to both climate change and changes in land elevation. To evaluate these different components, a satellite altimetry time series representing absolute sea level was used in conjunction with measurements of vertical land movement from Global Positioning System (GPS) locations in Phuket.

Satellite altimetry for the nearest location (7°N 98′E, approx. 89 km from the site) was extracted from the University of Colorado ‘sea level wizard’ at http://sealevel.colarado.edu ^21^. The time series (commencing in 1993) was examined for linear trends using generalised least-squares (GLS) regression, fitting autoregressive models to the data and selecting the best model using Akaike Information Criterion (AIC). Trend analysis was run in R 3.4.2^18^ using the package nlme^19^. Time series decomposition was also used to identify the main changes in sea level over time (Seasonal and Trend decomposition using Loess [STL]) with a seasonal period of 1 year and span of 1.5 years^22^.

#### Vertical land movement

GPS data from three (2 continuously and 1 yearly re-occupied, the latter located on bedrock and re-observed with the same antenna type and fixed height) locations in Phuket (10–35 km from the study area) were combined to provide a time series of ~11 years commencing at the end of 2000 until 2011 (see Supplementary Information and Fig. S2 for location of GPS sites).

Data (24 hour GPS measurements sampled at 30s intervals) for each location were analysed using GIPSY-OASIS II software in Precise Point Positioning mode^23^. The daily 3-dimensional station absolute positions were derived in the updated International Global Navigation Satellite System Service realization (IGS) of the International Terrestrial Reference Frame 2008^24^ (IGb08^25^) after which weekly averaged positions were computed. This averaging was performed to screen for any outliers and thereby improve the reliability of the coordinate solutions. Since the GPS observations at these 3 station locations overlap, it was possible to derive a single position time series for the vertical position component by reference to a common baseline. The vertical time series was fitted with a seasonal variation^26^ and modelled as A*sin(α) + B*cos(α), where α is 360°/365.25days * (time in days since first data epoch in late 2000). Parameters A and B were estimated along with the linear and non-linear regression functions.

The period of observations included the occurrence of the 2004 Mw 9.2 Sumatra Andaman earthquake. This resulted in instantaneous wide-spread horizontal co-seismic deformations in SE Asia which were also present in the GPS records from Thailand^27^. The earthquake has been followed by post-seismic deformations^28^ which differ significantly from the inter-seismic rates that were observed prior to the event^29,30^.

### Local reef characteristics, long-term monitoring of coral community structure and bleaching incidence

The reef flats in the study area are moderately diverse with over 30 scleractinian corals species^13^ and are dominated by massive coral species with occasional colonies of branching *Acropora*, *Pocillopora* and *Montipora* between them. Three sites on the reef flats, A, B and C (Fig. S1), have been monitored at least every 3–4 years over the period 1979–2018 using permanently marked 10 m long transects placed parallel to the shore at 10 m intervals on a line perpendicular to the shore to provide measures of coral cover and diversity by plotless line techniques (Supplementary Information for description of monitoring dates and sampling regime). Non-parametric multivariate analyses^31^ of transect data were applied using the package PRIMER v7^32^. Percent cover data of coral species along each transect were square-root transformed to down-weight the contributions of the most dominant species and then averaged within surveys. Bray-Curtis similarities were calculated and ordinated using non-metric multidimensional scaling (nMDS). To identify groups of species which varied coherently across surveys at each site we used Type 3 Similarity Profiles analysis^33^. For each site percent cover data of each species was standardised (converted to a percentage of the total cover of that species across all surveys) and the values used to calculate an Index of Association among species. The matrix of associations was clustered using hierarchical agglomerative clustering with group-average linkage. The associations among groups of species clustered at each node of the dendrogram were tested for multivariate structure using Similarity Profiles analysis. If the null hypothesis of ‘coherent species responses’ could not be rejected (using 9999 permutations and p = 1%) it was concluded that species grouped at that node in the dendrogram were varying coherently.

The sites have also been mapped using standard land-based surveying techniques (levelling and triangulation) to a common datum point^34^. Levelling records were taken at 5 m intervals (accuracy ± 3 mm) along lines perpendicular to the shore on which transects were placed (Supplementary Information Fig. S3) and reveal that the inner-mid reef flat at Site C is ~10 cm lower than that at Sites A and B while the outer reef flat at Site C is at least 20–30 cm lower than those at Sites A and B. As a result the timing and extent of aerial exposure differ between sites and photographic records and videos show the reef flat at Site C being uncovered 45 minutes later and covered 45 minutes earlier than that at Site B during low spring tides.

In addition to the above surveys, four permanently marked belt transects (10 m long × 60 cm width) on the outer reef flat at Site A have been photographed at least annually between 1987–2009, apart from 1989. In bleaching years the belt transects were photographed at least twice following the initial bleaching. These four transects (9, 11, 14 and 15) are parallel to the shore and are 90, 110, 140 and 150 m, respectively from the shore line. Using the photographic-transects, 64 colonies of a selection of massive coral species were repeatedly assessed for bleaching incidence in photographs taken at peak bleaching times in 1991, 1995, 1998, 2003, 2010 and 2016. Colonies were assessed as either partially bleached or fully bleached using established reference standards supported by zooxanthellae counts (see Supplementary Information for details). Coral cover data from these transects^14^ were also analysed in R 3.4.2^18^ using time series decomposition to identify changes in coral cover over time (Seasonal and Trend decomposition using Loess [STL]) with a seasonal period of 1 year and span of 25 months^22^. This additional coral cover data set from Site A provided a higher resolution, in terms of regularity of monitoring, than the detailed transect information gathered from reef sites A, B and C described above.

## Results

### Environmental factors

#### Sea surface temperature

Sea surface temperatures at the study location have been rising at a rate of 0.159 ± 0.03 °C (mean ± SE) per decade (p < 0.0001, GLS AR3) over the period 1979–2017 (Fig. 1). Marked thermally-induced coral bleaching was noted on the shallow reef flats in 1991, 1995, 1998, 2003, 2010 and 2016 when sea temperatures exceeded the putative temperature threshold for bleaching of 30.1 °C described earlier^4^. High temperatures in other years resulted in either no or very limited bleaching of corals on the intertidal reef flats. The most extreme bleaching event on record occurred in 2010 when sea temperatures exceeded 31 °C for over 7 weeks. Degree Heating Weeks in 2010 were also the highest on record and exceeded those in 2016 when equally high SSTs were restricted to only 3–4 weeks.

#### Sea-level rise

**Figure 2:**
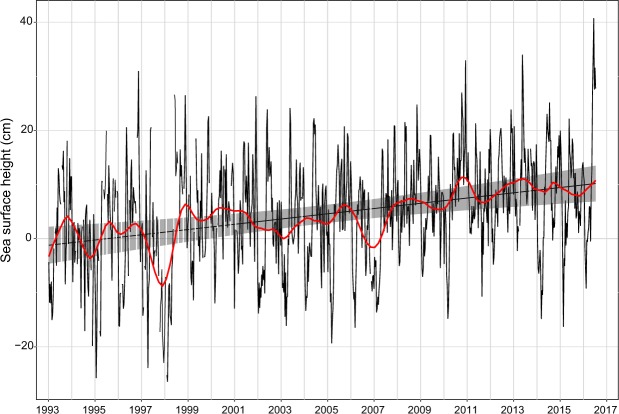
Satellite altimeter sea surface height anomalies for Phuket (98°E 7°N). Linear trend line (slope 4.85 ± 1.262 mm y^−1^ (mean ± SE) fitted by GLS AR4 model (p < 0.0001) with 95% confidence intervals (grey shading) and STL Loess trend (red). The full STL decomposition plot is shown in Supplementary Fig. S4. Periods of missing data shown by white vertical lines on the confidence intervals.

Mean sea level at Phuket has a marked seasonal component which results in a 20 cm variation between the dry season (December-April) and the wet season (May-November)^14^. In addition, there may be larger variations of up to ±46 cm (including seasonal variation) which are linked to Indian Ocean Dipole (IOD) phenomena^14^. Positive IOD events lead to depressed sea levels around Phuket and have occurred regularly in recent years. One of the most pronounced events took place in 1997–98 when sea levels were depressed over a 7 month period from September 1997- March 1998 with a maximum sea-level depression of 29 cm which is revealed in the satellite altimetry plot (Fig. 2) for the region.

The satellite altimetry data (Fig. 2) can be fitted by a linear model which gives a rate of rise in sea level of 4.85 ± 1.262 mm y^−1^ (mean ± SE) (AR4 model p < 0.0001) over the period 1993–2016. Time-series decomposition also helps to reveal periods of positive and negative sea level anomalies. At the start of the time series sea-level anomalies were largely positive, followed by the large 1997–98 sea-level depression. Thereafter there were no marked negative anomalies until the positive IOD of 2007–2008 when sea levels, although lowered, were not as severely depressed as in 1997–98. Post-2008, sea-level anomalies appear to have been generally positive until the end of the time series in mid-2016.

#### Vertical land movement

Previous GPS results in Thailand showed that the island of Phuket is located at the edge of a plate boundary deformation zone which has displayed significant deformations prior^25^, during^23^ and after^24,35^ the 2004 earthquake.

**Figure 3:**
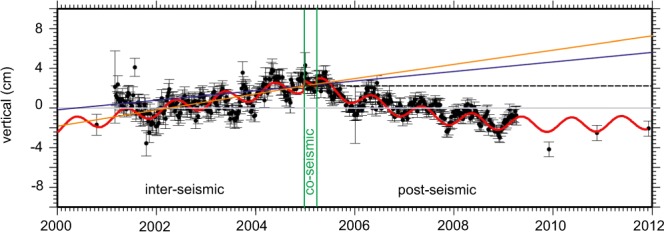
Combined station GPS vertical position time series for Phuket (black data points ± SE). The vertical height reference point (indicated by the dashed black line) is the computed averaged position just before the Mw 9.2 earthquake (26 December 2004) which initiated significant (post-seismic) changes in the vertical position. The red line is the fitted trend that takes into account a linear inter-seismic phase motion, co-seismic phase (instant) position jumps (2004/2005) and post-seismic phase non-inear motion. The blue line represents the satellite altimetry linear trend from Figure 2 (4.85 ± 1.262 mm y^−1^ (mean ± SE)) and the orange line is the inter-seismic period velocity estimate (7.19 ± 2.55 mm y^−1^ (mean ± SE)). The relative sea-level change over the period 2004–2011 is thus represented by the vertical distance between the red and blue lines (~77 mm) on the right hand axis, and the corresponding vertical land subsidence is the distance between the red and dashed line on this axis (~44 mm).

The combined GPS record (Fig. 3) from late 2000–2011 confirms and extends these earlier published results and shows the transition from inter-seismic to post-seismic motion after an almost instantaneous (small, <10 mm) co-seismic shift in the vertical position of the island. While the inter-seismic motion trend appears quasi-linear (7.19 ± 2.55 mm y^−1^ (mean ± SE)) along with typical seasonal annual variations, the post-seismic deformation phase follows an (approximately) exponential decay pattern. This pattern is expected to become linear once the post-seismic cycle phase has ended, but this may take decades and geophysical models are not yet able to accurately predict when this may occur.

The changes in the absolute vertical position are clearly visible, and by the end of 2011 Phuket was located 43.73 ± 8.06 mm (mean ± SE) lower than it was before the earthquake (i.e. a current rate of ~6.3 mm y^−1^). This is a significant short-time change in the vertical motion of the island.

Taking into account the absolute sea-level change estimate of 4.85 mm y^−1^ observed by satellite altimetry (Figs 2, 3 blue line) and the observed inter-seismic land motion (+7.19 mm y^−1^) it appears that relative sea-level changes would have been neutral to slightly negative up to the Mw 9.2 event, i.e. the island was being uplifted equally or slightly more than sea-level rise in the Andaman Sea.

This inter-seismic uplift is also known to have been persisted prior to 2000. Earlier analysis of a tide gauge record from Ko Taphao Noi (1.5 km from the study area) provides corroboration for both the uplift and its long-term nature^14^. In that analysis, no signal of sea-level change was found for the period 1940–2010 or for a shorter period paralleling the satellite altimetry record 1993–2010. The tide gauge record has not been used in the present study because of unresolved datum problems in the recent record which may partly be due to a refurbishment in 2013–15 when a new tide gauge platform was constructed.

After the Mw 9.2 earthquake, while absolute sea-level rise continued, the island has been subsiding in a non-linear fashion (the latest and previous GPS results suggest an average land fall of 6 mm y^−1^ (2005–2011) to 12 mm y^−1^ (2005–2009)^35^) due to the geophysical processes associated with the post-seismic earthquake phase. As a result, the combination of land movement and satellite altimetry indicates that since the end of 2004, the relative sea-level in Phuket has increased non-linearly by 77.41 ± 11.91 mm in just 7 years, equivalent to a linear annual rate of 11.15 ± 1.71 mm y^−1^ (Fig. 3).

### Incidence of bleaching, temporal changes in coral cover and coral community structure

Table 1 shows the incidence of bleaching in selected coral colonies on permanent photo-transects during the peak bleaching period of each of the six years when major coral bleaching occurred. Lowest levels of bleaching were observed in 1998 and 2003 with similar numbers of colonies showing total and partial bleaching. Higher levels of overall bleaching were observed in 1991, 1995 and 2016 although the proportion of totally and partially bleached corals varied between years. Outstanding in the data series is 2010 where 91% of colonies were recorded as totally bleached and 7% were partially bleached, resulting in an overall bleaching incidence of 98%. In 1991 and 2016 the incidence of bleaching was also high, but in contrast to 2010, the majority of corals were partially bleached with relatively few colonies assessed as totally bleached.

**Table 1:** Incidence of percentage bleaching in massive coral colonies at Site A (n = 64) from permanently marked photo-transects during major coral bleaching events.

Year	1991	1995	1998	2003	2010	2016
% totally bleached	18	37	7	10	91	16
% partially bleached	55	30	36	29	7	54
% overall bleaching incidence	**73**	**67**	**43**	**39**	**98**	**70**

**Figure 4:**
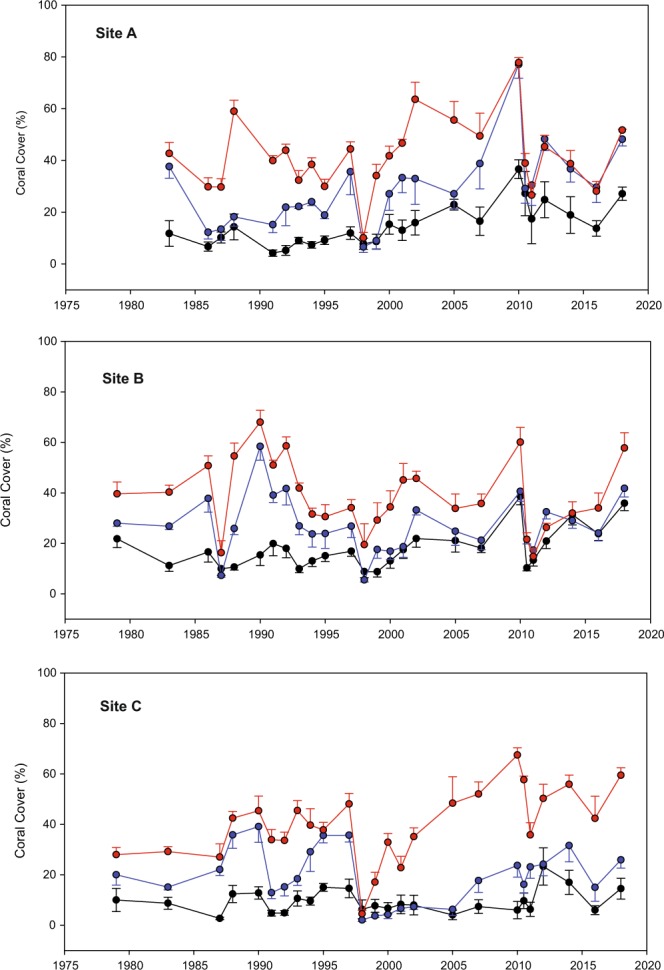
Plots of average coral cover (±SE) over time on inner (black), mid (blue) and outer (red) reef flats at Sites A, B and C.

Plots of mean coral cover (±SE) at all three sites on inner, mid and outer reef flats over the study period reveal considerable temporal and spatial variation (Fig. 4). In terms of coral cover the outer reef sites appear to be most sensitive to environmental changes such as the dredging event in 1986–87 (evident at Sites A and B but not at Site C which was more distant from the impact), the temporarily lowered sea level in 1997 (evident at all sites), relative sea level change (neutral prior to 2004, rising thereafter) (evident at all sites), and the thermal bleaching event in 2010 (most evident at Sites A and B). A steady recovery from the 2010 bleaching event is obvious at all sites over the period 2010–2018. Interestingly, the earlier bleaching events appear to have had relatively little influence on coral cover though in 1991 cover declined on the outer reef at Site A, the mid and outer reef of Site B and the mid reef at C. However, repeated permanent transect photography and coral chronology reveal that this coral mortality was associated with aerial exposure and solar bleaching of highly elevated corals due to lowered sea level in 1990–91^36^ rather than to thermally–induced bleaching. The 2016 thermally-induced bleaching event did not have a major impact on coral cover and subsequent recovery at any site.

**Figure 5:**
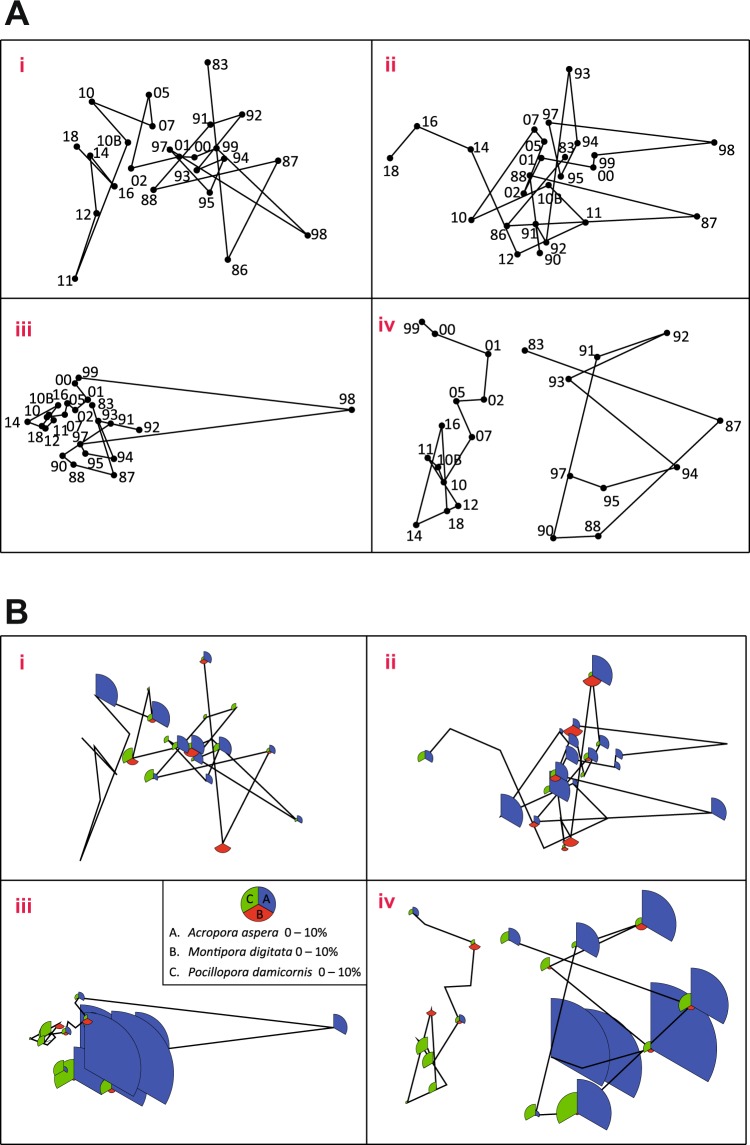
(**A**) Ordination by non-metric multidimensional scaling (nMDS) of Bray Curtis similarities among averaged coral species level cover within site-year combinations. Calculations used average square-root transformed present cover data from **i**) Site A (2d stress = 0.15); **ii**) Site B (2d stress = 0.14); **iii**) Site C (2d stress = 0.09); and **iv**) Site C omitting data from 1998 (2d stress = 0.13). Labels indicate years 1983–2018; 10B indicates repeat survey undertaken during month of peak bleaching in 2010. (**B**) Ordination (nMDS) as in **A** overlaid with representations of branching coral species abundance. Plots of **i**) Site A, **ii**) Site B **iii**) Site C and **iv**) Site C omitting 1998 with overlaid bubbles scaled in size in proportion to the total percent cover of three branching species namely *Acropora aspera*, *Montipora digitata* and *Pocillopora damicornis* in each survey (see key).

Non–metric MDS analysis of the data set from each site showed major disturbances to the community structure in those years when there have been extreme environmental influences (Fig. 5A), namely the dredging in 1986–87, the lowered sea level of 1997–98, and then the bleaching in 2010 where effects were particularly marked at sites A and B by 2011. The level of disturbance to the reef community varied at each site with one of the most pronounced effects being the sea-level depression in 1997–98 at Site C. Here, following this event, there was a major switch in the community structure of the reef which has not returned to its former state some 20 y later. Reasons for this change can be seen in the MDS plot shown in Fig. 5B where extensive branching *Acropora aspera* cover (~40%) at this site almost disappeared following the extended period of low sea-level. Branching corals have failed to recover at this location because the mobile rubble generated by the coral mortality has proved to be an unsuitable substrate for settlement of coral recruits.

At Sites A and B the appearance of branching species has been sporadic over time but *A. aspera*, in particular, became evident in the early 2000s reaching its maximum coverage in 2010. Following the extensive bleaching in 2010 this species disappeared from monitored transects at these sites but re-appeared on the outer reef flat at Site B by 2018 and also at Site A as isolated colonies that were not present on the transect lines (B. Brown – pers. obs.) At Site C, where the effects of bleaching were not so marked, branching cover since 1998 has been dominated by *Pocillopora damicornis*, though isolated colonies of *A. aspera* were also noted on the outer reef flat in 2018 but were again absent from monitored transect lines (B. Brown – pers.com). The branching coral *Montipora digitata* has shown no marked pattern of abundance over time at any site.

**Figure 6:**
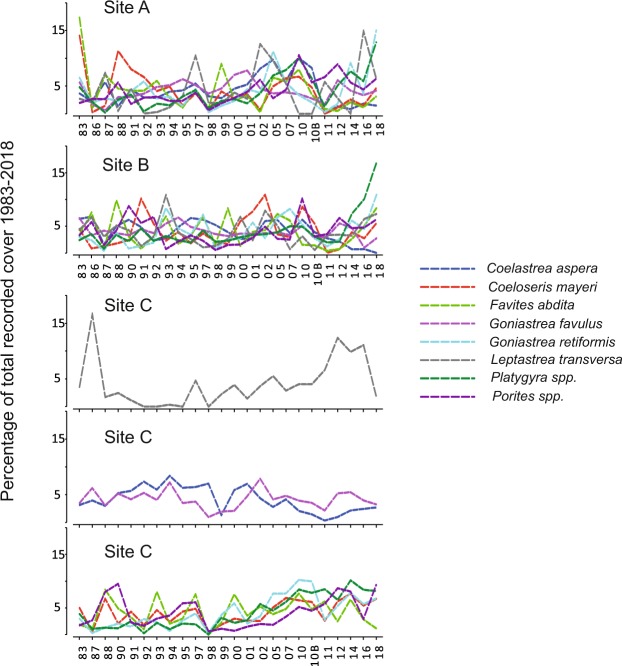
Coherence plots for massive coral species at Sites A, B and C using Similarity Profiles analysis. Percent cover for each species is rescaled as the proportion of total cover recorded for that species in all surveys, to focus on ‘similarity in species’ variation among surveys through time rather than on differences in relative cover. Species grouped within individual graphs vary coherently across surveys at each site.

Temporal patterns of species as revealed through Similarity Profiles analyses show the variation of distinct sub-sets of species across surveys as component line plots (coherent curves). Analyses at each site show that while branching species tend to come and go, there is a group of massive species that tend to vary as a coherent group. At Sites A and B all the major massive species co-vary over time (Fig. 6). These species constitute up to 90% of total coral cover and it would appear that no particular species became dominant immediately after the 2010 bleaching. At Site C the suite of massive species that vary coherently include the mid-outer reef flat species *Coeloseris mayeri*, *Favites abdita*, *G. retiformis*, *Platygyra spp*. and *Porites spp*., while the inner reef flat *C. aspera* and *Goniastrea favulus* display a significantly different pattern, as does *Leptastrea transversa*. The different sub-sets of species defined by the Similarity Profiles Analysis at this site, compared to Sites A and B, underline how differing environmental conditions, no doubt dictated by the greater water depth, influence species associations and zonation patterns.

## Discussion

Sea temperatures around Phuket have been rising over the last four decades and are currently ~0.6 °C warmer on average than in 1979 when our study first began. Early predictions for this location that increased temperature anomalies over time would lead to enhanced bleaching-induced mortality^4^ appear to be borne out, at least in part, with the 2010 bleaching being the most damaging on record. However, although sea temperatures were equally extreme in 2016 and the incidence of bleaching was high, the resulting effects on coral cover and community structure were negligible. A reason for this is likely to be that in 2016 SST dropped in late May unlike 2010 when SST exceeded the putative bleaching threshold for up to 4 months from March to the end of June^37^. Thus in 2010 several massive coral species remained bleached for up to 15 weeks after initial bleaching before ensuing mortality^38^ whereas repeated photography of permanent transects in 2016 showed that these same species recovered their colour some 6 weeks after initial bleaching and subsequently survived.

Interestingly, the recent bleaching years of 1998, 2003, 2010 and 2016 have all been characterised by a late onset of the Bay of Bengal Summer Monsoon (BoBSM)^39^. The timing of the onset appears to be significantly affected by ENSO with an advanced/delay in onset following a cold/warm ENSO event^39^. Such a delay in a warm ENSO year is critical for corals in the Andaman Sea as it results in the late arrival of wet and cloudy conditions which normally lower SST in the region and provide some respite for corals living at the limits of their temperature tolerance. Modelling of the effects of global warming on the monsoon onset suggest dates in future will either be delayed or show no change but confidence in these results is limited due to the inability of the models to reproduce the monsoon climate and the large scatter in model projections^40^.

Concomitant with long-term increases in SST in the region has been an increase in sea level due to climate change. Satellite altimetry shows a linear rise in absolute sea level of 4.85 mm y^−1^ (1993–2016), a rate which may have increased from earlier estimates for the period 1993–2010^14^ (3.37 mm y^−1^). In addition, it is also now apparent that vertical land deformation is a critical factor affecting sea-level changes at Phuket since the island borders an extremely active tectonic region. From at least late 2000 until 2004 vertical land motion was positive (7.19 mm y^−1^) and as a consequence relative sea-level change was largely negative or neutral, with land uplift exceeding absolute sea-level rise. Following the 2004 Sumatran earthquake, however the pattern has reversed and the land at Phuket has been subsiding at an estimated rate of between 6–12 mm y^−1^ over the period 2005–2009/2011^35^.

**Figure 7:**
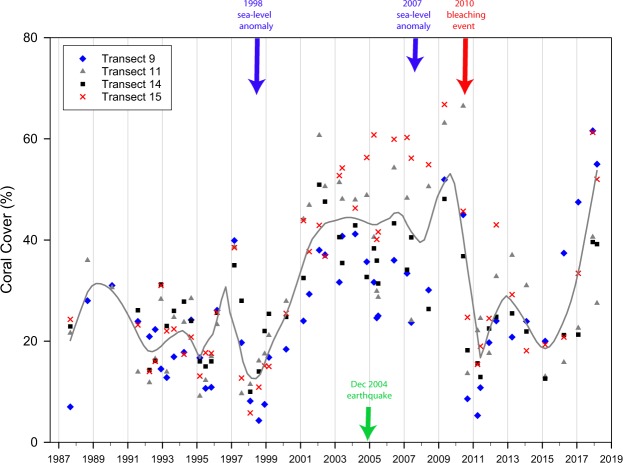
Live coral cover (%) on the permanent belt transects at Site A displayed as individual sampling points for each of the 4 transects. The dark grey line represents STL trend with a span of 25 months. The full STL decomposition plot is shown in Supplementary Fig. S5.

Our earlier work^14^, using coral cover data from permanent photo-transects at Site A, demonstrated that reef flat corals at our study area benefitted from periods of elevated sea level, with live coral cover positively correlated with mean sea level as experienced over the preceding months (between 6–16 months). Over the period 1998–2010 we reported significant increases in coral cover over time (Fig. 11 in^14^) and hypothesised that this was driven by a small increase in relative sea level over the period 1960–2009 (2.7 mm y^−1^). Although at the time we were aware of land subsidence following the 2004 earthquake we did not have evidence of land elevation changes in the inter-seismic period. Revisiting our earlier work and updating our analysis of coral cover over time, we can now show that from the beginning of our data set in 1987 until the end of 2004 during the period when relative sea-level rise was neutral, coral cover fluctuated between ~10–30% (Fig. 7). The marked negative IOD sea-level anomaly in 1998 then caused a drop in coral cover followed by a sustained period of recovery when sea-level anomalies were positive (Fig. 2). In 2007 a smaller but nonetheless sizeable negative anomaly again led to a reduction in coral cover although by now the sustained period of relative sea-level rise had commenced following the 2004 earthquake. A dramatic fall in coral cover in 2010 followed the bleaching event in that year. Thereafter, the influence of rising relative sea level of ~11 mm y^−1^, and a lack of major negative sea-level anomalies can be seen to stimulate the recovery of coral cover again.

The mechanisms underlying the observed increase in coral cover with increased sea level is likely to be the associated increase in ‘accommodation space’ allowing for expansion of reef corals in both lateral and vertical dimensions^14,41–43^. However, as these studies also highlight, the impact of other factors such as rising background SST and positive temperature anomalies may limit the ultimate ability of individual corals to keep pace with sea-level rise as a result of damaging bleaching events and decreased calcification rates of dominant species^44^.

In the current study we have had the opportunity to evaluate the effects of the most extreme bleaching event on record during a period of rapid sea-level rise with particular reference to coral cover, community changes and bleaching recovery rates. Although the 2010 bleaching event caused a loss of live coral cover in a common suite of massive species at all sites, reef responses have shown considerable spatial differences. The most adversely affected reefs were at Sites A and B while Site C showed lower losses in coral cover and minimal disruption to overall community structure. A combination of factors is likely responsible for this nuanced result. For example, at Site C compared to Sites A and B, the reef flat is deeper, leading to decreased periods of aerial exposure, and the reef profile is steeper which allows rapid flushing of seawater on and off the reef. In addition, bleaching susceptibility of submerged corals at Site C during low tides would be lowered as a result of increased water flow^45^ and a reduction in damaging irradiance/SST interactions^46^ by the highly turbid overlying waters^47^.

Reef recovery from the 2010 bleaching, in terms of coral cover has taken approximately 8 years at our most damaged sites. Considerable changes in community composition associated with the event were visible at Sites A and B, and less so at Site C. Species composition at Sites A and C rapidly returned to a situation similar to that prior to the event, though the continuing changes in composition at Site B suggest either that recovery is still ongoing or that the community is undergoing a transition to an alternate state. Recovery times for reefs suffering from severe bleaching at sites worldwide are extremely variable depending on disturbance severity, reef location and setting and coral community structure. Shallow reef flats in the Ryukus, Japan dominated by branching *Montipora* (40% cover) took only 2 years to recover from the severe bleaching of 1997–98^48^. *Montipora digitata* on another shallow reef flat in Okinawa in the same region was significantly negatively affected by the 1997–98 event in the short term but after 10 years it was considered a long-term winner, having doubled its pre-bleaching cover^49^. In Okinawa, while species richness recovered after 10 years the reef composition had changed though many of the massive *Porites* species, and merulinids survived the earlier severe bleaching. Much longer recovery periods have been noted in other studies such as in the Persian/Arabian Gulf where modelling suggests that a period in excess of 15 years might be required for full recovery of the reef assemblage^50^. On the Great Barrier Reef where recovery rates from chronic stresses, that included heat stress, were compared pre- and post-2002 the ability of corals to regain cover during disturbance free intervals has been reduced in recent years^51^. Similarly in the Lakshadweep archipelago, in the central Indian Ocean, coral recovery rates from recent bleaching events have decreased^52^ mainly as a result of the demise of fast growing branching species and increasing dominance of slower growing massive corals.

Recovery of the reef flats at Phuket from the 2010 bleaching has been relatively rapid given that background rising SST has also reduced massive coral growth rates at these sites in recent years^44^. Dramatically lowered coral growth rates induced by the bleaching in 2010^53^ and longer-term growth effects continuing well beyond the event^54,55^ would also likely contribute to the length of time needed for recovery. Although many of the massive corals dominating the reef flats at Phuket are thermally tolerant they suffered considerable partial mortality as a result of the bleaching in 2010 with recovery involving tissue overgrowth of recently dead areas of the colony. Such recovery mechanisms have been noted previously in these corals as a result of other disturbances such as sub-aerial exposure^36^. In some massive coral species total colony mortality followed the bleaching and this response was particularly evident in *Coelastrea aspera* at Sites A and B where the largest and oldest corals showed a marked decline by 2011^56^. Although juvenile corals survived^57^ *C. aspera* has yet to return to pre-bleaching cover values at these sites.

Overall, the relatively rapid recovery of the majority of slow growing massive corals, its limited effect on the lowest lying reef site, and the continued recovery of all reef sites during a second extreme but short-lived bleaching in 2016 suggests that rising sea level not only offered increased ‘accommodation space’ to corals up to 2010 but may also have reduced the damage from the thermal bleaching in 2016 through increased water depth (reduced light intensity and reduced sub-aerial exposure)^46^. Acclimatisation of corals to bleaching drivers (high SST and irradiance) through experience of earlier bleaching events may also have played a positive part in recovery with environmental ‘memory’ of defences against factors such as high irradiance and elevated SST lasting at least 10 years^56^.

While we have considered above the short-term benefits of rising sea level on the intertidal reef flats, negative effects of rapid sea-level rise on coral reefs have been widely reported^3,58^ and future climate scenarios may see reefs affected by severe bleaching unable to maintain their vertical growth^3^. The carbonate budget of the macro-tidal reef flats at Phuket has been calculated as at least 3 kg CaCO_3_ m^−2^ y^−1^ based on the calcium carbonate production of five dominant genera^59^. This value is relatively high both for the Indian Ocean^3^ and also for a reef flat setting where calcification rates are generally lower than the shallow reef front^43^.

The reef flats of Phuket could be described as coral reef ‘oases’ which are characterised by their ability to both resist and rebound from disturbances^60^. Similar reef settings, with an estimated area in excess of 900 km^2^, are found throughout the Andaman Sea^61^ and along the Kimberley coast of north west Australia^62^ offering the potential of increased reef resilience in the eastern Indian Ocean. The future of reefs in the Andaman Sea will ultimately be determined by the rate of relative sea-level rise and the ability of corals to keep pace with this increase which in turn will be influenced by the intensity and frequency of bleaching events, the timing of BoBSM onsets and IOD-related sea-level depressions. However, in the short term the positive role of rising sea level on these coral reefs should not be under-estimated.

## Supplementary information

Supplementary Information

## Data Availability

The coral datasets generated and/or analysed during the current study are publicly available online from the University of Newcastle Research Repository at http://data.ncl.ac.uk.
