# Supplementary material for: Long-term impacts of rising sea temperature and sea level on shallow water coral communities over a ~40 year period
Source: Sci Rep. 2019 Jun 19;9:8826. doi: 10.1038/s41598-019-45188-x (PMC6584745; doi:10.1038/s41598-019-45188-x)
Supplement: Supplementary file 1 — Supplementary Information [file 41598_2019_45188_MOESM1_ESM.docx]

Supplementary Information

Title**: Long-term impacts of rising sea temperature and sea level on shallow water coral communities over a ~40 year period**

B. E. Brown^1,2,^ **^*^**, R.P. Dunne^3^, P.J. Somerfield^4^, A.J. Edwards^1^, W.J.F. Simons^5^, N. Phongsuwan^6^, L. Putchim^7^, L. Anderson^8^ & M.C. Naeije^5^

^1^School of Natural and Environmental Sciences, Newcastle University, Newcastle upon Tyne NE1 7RU, UK.^2^Environmental Research Unit, University of the Highlands and Islands, Castle Street, Thurso, Caithness KW14 7JD, Scotland, UK. ^3^West Briscoe, Baldersdale, Barnard Castle, Co. Durham DL12 9UP, UK. ^4^Plymouth Marine Laboratory, Prospect Place, West Hoe, Plymouth PL1 3DH, UK. ^5^Department of Space Engineering, Delft University of Technology, Netherlands. ^6^Department of Marine and Coastal Resources, 120 Moo 3, Changwathana Road, Bangkok,10210, Thailand. ^7^Phuket Marine Biological Center, PO Box 60, Phuket 8300, Thailand.^8^Faculty of Biological Sciences, University of Leeds, Leeds, LS2 9JT, UK.

**^*^** corresponding author: e-mail ProfBarbaraBrown@aol.com

**Methods**

Study Site


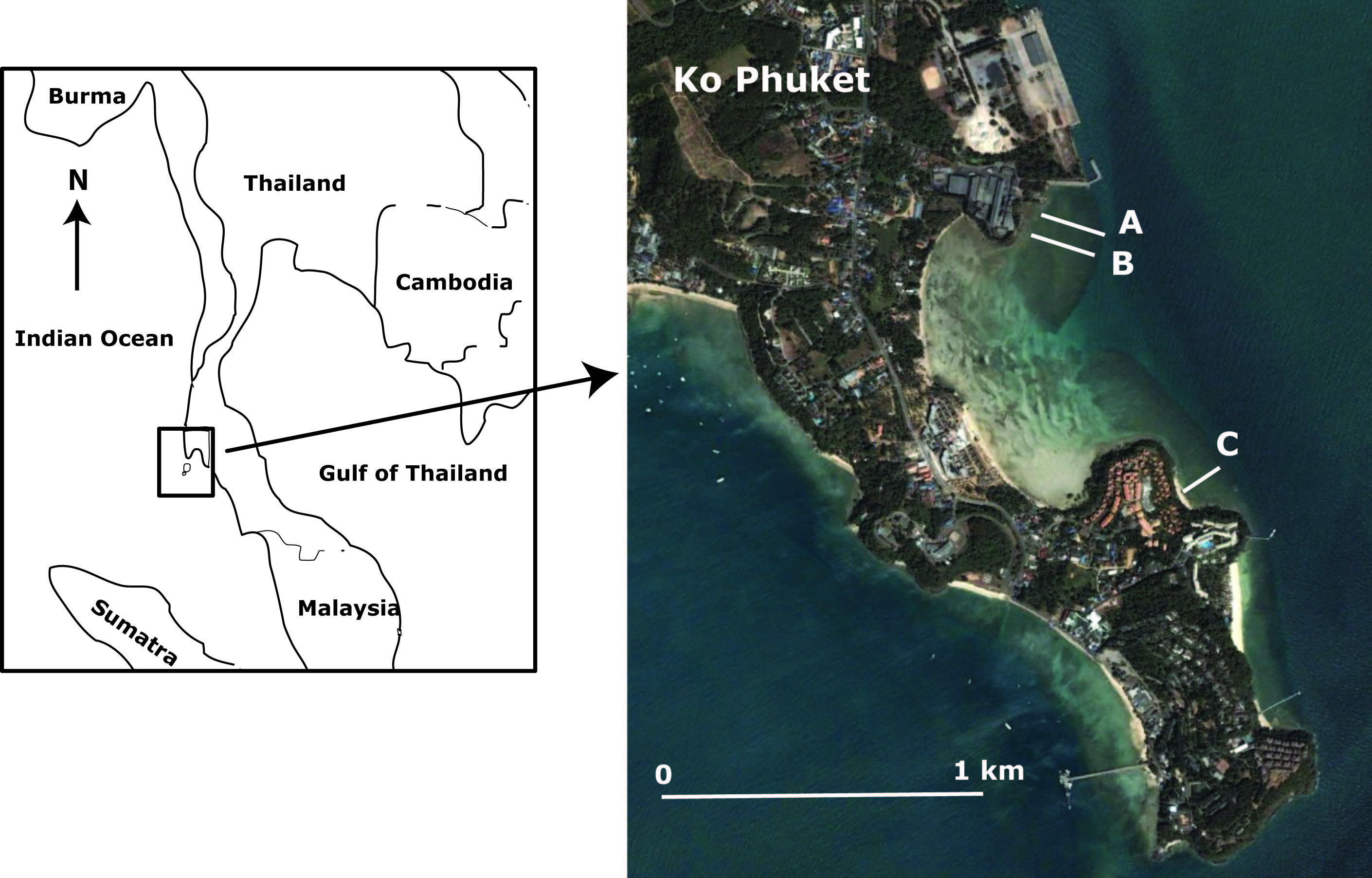


**Figure S1** Map showing location of Sites A, B and C on the south-east tip of Phuket, Thailand. Satellite imagery (dated 18 Jan 2009) courtesy of Google Earth (© 2011 Digital Globe)

Vertical land movement

Geodetic GPS data suitable for scientific processing and yielding high precision position results with millimetre accuracy in Phuket were first available during the Geodynamics of South and Southeast Asia (GEODYSSEA) 1994-1998 project. PHUK is one of ~40 GPS campaign sites (located on bedrock) that make up the GEODYSSEA network in Southeast Asia (Fig. S2). This network was designed to accurately determine the Sundaland plate motion and plate boundaries and verify whether it is currently moving independently from the Eurasian plate. The PHUK point has been observed in campaign style (3-5 days) almost annually by the Royal Thai Survey Department (RTSD) since 2000 after the GEODYSSEA project ended, and even more frequently in the years following the Mw 9.2 Sumatra-Andaman earthquake. Additionally, data was used from two continuous GPS stations operational in Phuket, named PHKT (from 2000-2007) installed as part of the Japanese GAME-T station network and the PTAC (also known as PTCT/PTC1) (from 2006-2012), a station managed by the National Institute of Information and Communications Technology (NICT) of Japan.


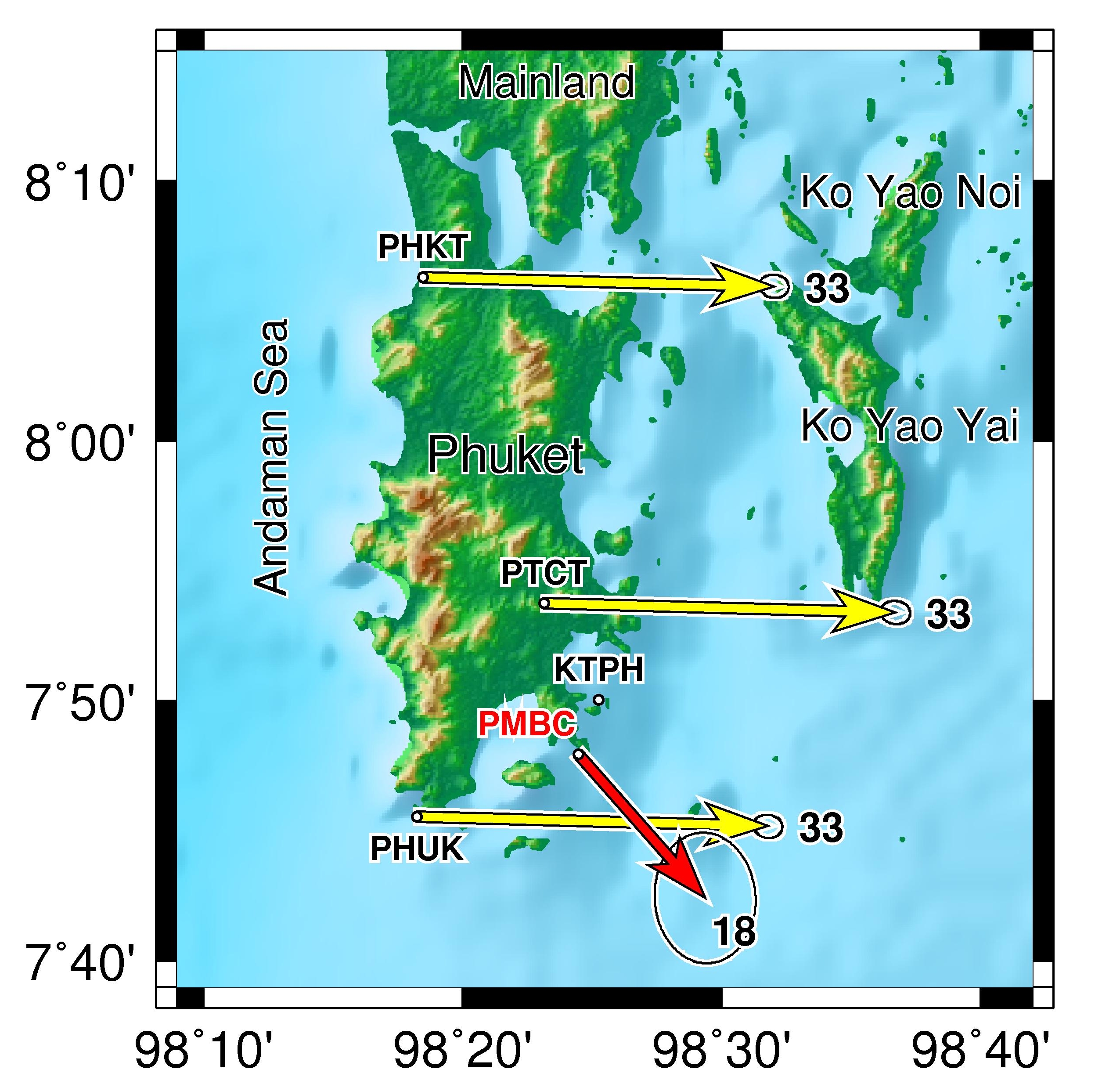


**Figure S2** Location of the 3 GNSS stations (PHUK, PHKT, PTCT) in Phuket Island along with their (in mm y^-1^) absolute GPS station (inter-seismic) velocities (in yellow)^1^ while the red arrow represents the current ongoing (post-seismic) motion of the new station PMBC (2017-present) on Phuket Island (Simons –pers com). Error ellipses show 95% confidence levels.

Coral Community Analyses

Line transects at Sites B and C were monitored during the dry season (November-April) in the majority of years (1979, 1983, 1986, 1990, 1992, 1993, 1994, 1995, 1996, 1997, 1998, 1999, 2000, 2001, 2002, 2005, 2007, 2010, 2011, 2012, 2014, 2016 and 2018). For Site A monitoring began in 1983 and was repeated in the years described for Sites B and C. In the bleaching years of 1991, 1995, 1998 and 2010 surveys were also carried out in June-July post-bleaching. For each line transect all conspicuous benthic lifeforms underlying the 10 m long transect line were monitored but since cover by organisms other than corals (e.g. macro-algae, soft corals, ascidians and sponges) constitute <1% cover we refer only to cover of scleractinian coral species. Measured parameters include percentage cover of each species and total number of colonies of each species. Transect lines were laid parallel to the shore from the shoreline to the outer reef edge so encompassing inner, mid and outer reef flat positions. Site A comprised 12 x 10m long transects, Site B 17 x 10m transects and Site C 12 x 10m transects.

Four 10 x1m permanently marked belt transects at Site A were photographed annually during February-March each year from 1983-2018 (apart from 1989) and in the bleaching years of 1991, 1995, 1998, 2003, 2010 and 2016 were re-photographed in both July and October. For bleaching assessments 64 colonies comprising the dominant massive coral species (e.g. *Coelastrea aspera*, *Goniastrea favulus*, *Goniastrea retiformis*, *Platygyra* spp, *Porites* spp., *Coeloseris mayori*) were repeatedly assessed during the peak bleaching period of bleaching years as either totally bleached or partially bleached. Total bleaching was ascribed to colonies that were overall white in colour (i.e. colour score 1 on coral colour reference card^2^) while colonies where only part of the colony was white were designated as partially bleached. Earlier work^3^ on the species selected had shown that colonies which were overall white had lost at least 80% of their normal algal complement during temperature-induced bleaching events.

Reef Flat Profiles


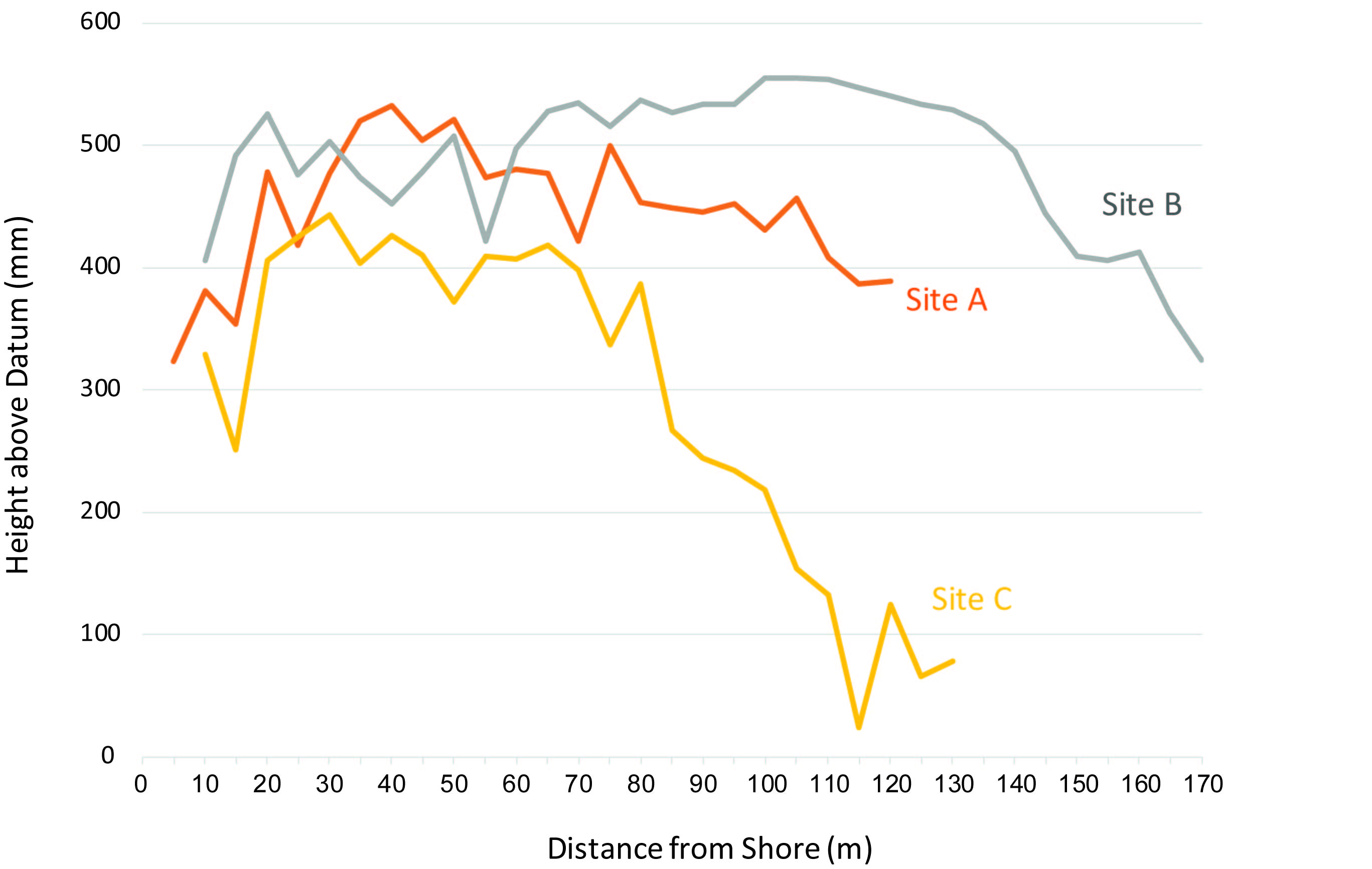


**Figure S3** Profiles of reef flats at Sites A, B and C relative to common datum linking all sites.

**Results**

Sea-level rise


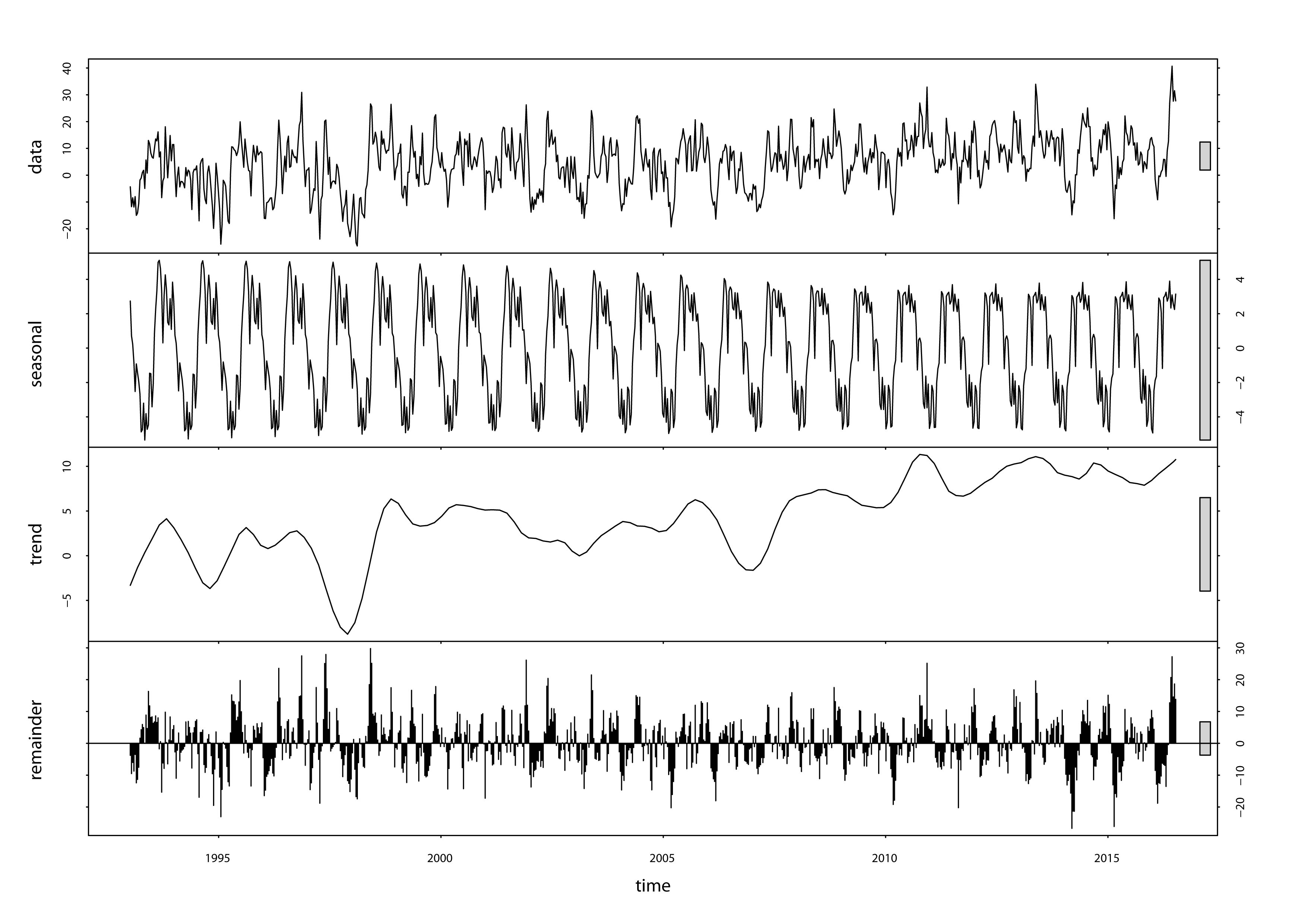


**Figure S4** STL decomposition plots for the satellite altimetry time series shown in Figure 2.

**Discussion**


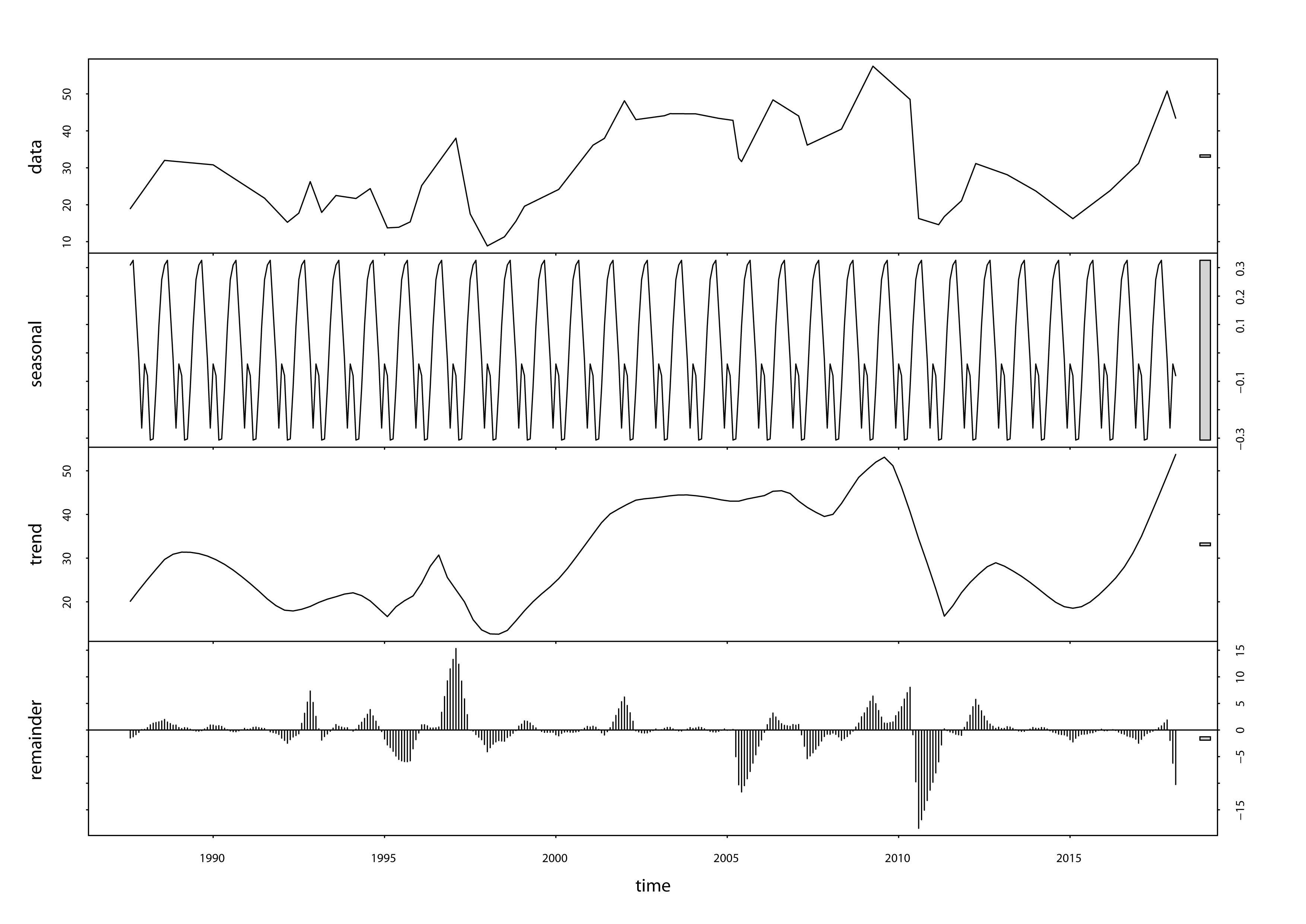


**Figure S5** STL decomposition plots for the live coral cover data shown in Figure 7.

**References**

^1^Mustafar, M.A., Simons, W.J.F., & Tongkul, F. Quantifying deformation in North Borneo with GPS. *J Geod*. **91**, 1241, [doi.org/10.1007/s00190-](https://doi.org/10.1007/s00190-)017-1024-z (2017)

^2^Siebeck, U.E., Marshall, N.J., Kluter, A., & Hoegh-Guldberg, O. Monitoring coral bleaching using a colour reference card. *Coral Reefs* **25**,453-460, doi. /10.1007/s00338-006-0123-8 (2006)

^3^Brown, B.E., Dunne, R.P., Ambasari, I., Le Tissier, M.D.A., & Satapoomin, U. Seasonal fluctuations in environmental factors and variations in symbiotic algae and chlorophyll pigments in four Indo-Pacific coral species. *Mar Ecol Prog Ser* **191**:53-69, doi. 10.3354/meps191053 (1999)
